# Development and validation of a multiplexed-tandem qPCR tool for diagnostics of human soil-transmitted helminth infections

**DOI:** 10.1371/journal.pntd.0007363

**Published:** 2019-06-17

**Authors:** Katharina Stracke, Naomi Clarke, Camille V. Awburn, Susana Vaz Nery, Virak Khieu, Rebecca J. Traub, Aaron R. Jex

**Affiliations:** 1 The Walter and Eliza Hall Institute of Medical Research, Melbourne, Victoria, Australia; 2 Faculty of Medicine, Dentistry and Health Sciences, The University of Melbourne, Melbourne, Victoria, Australia; 3 Research School of Population Health, Australian National University, Canberra, Australian Capital Territory, Australia; 4 Kirby Institute, University of New South Wales, Sydney, New South Wales, Australia; 5 National Center for Parasitology, Entomology and Malaria Control, Ministry of Health, Phnom Penh, Cambodia; 6 Faculty for Veterinary and Agricultural Sciences, The University of Melbourne, Melbourne, Victoria, Australia; Universite de Montreal, CANADA

## Abstract

Soil-transmitted helminths (STH) are a major cause of morbidity in tropical developing countries with a global infection prevalence of more than one billion people and disease burden of around 3.4 million disability adjusted life years. Infection prevalence directly correlates to inadequate sanitation, impoverished conditions and limited access to public health systems. Underestimation of infection prevalence using traditional microscopy-based diagnostic techniques is common, specifically in populations with access to benzimidazole mass treatment programs and a predominance of low intensity infections. In this study, we developed a multiplexed-tandem qPCR (MT-PCR) tool to identify and quantify STH eggs in stool samples. We have assessed this assay by measuring infection prevalence and intensity in field samples of two cohorts of participants from Timor-Leste and Cambodia, which were collected as part of earlier epidemiological studies. MT-PCR diagnostic parameters were compared to a previously published multiplexed qPCR for STH detection. The MT-PCR assay agreed strongly with qPCR data and showed a diagnostic specificity of 99.60–100.00% (sensitivity of 83.33–100.00%) compared to qPCR and kappa agreement exceeding 0.85 in all tests. In addition, the MT-PCR has the added advantage of distinguishing *Ancylostoma* spp. species, namely *Ancylostoma duodenale* and *Ancylostoma ceylanicum*. This semi-automated platform uses a standardized, manufactured reagent kit, shows excellent run-to-run consistency/repeatability and supports high-throughput detection and quantitation at a moderate cost.

## Author summary

Soil-transmitted helminthiases are among the most prevalent and damaging neglected tropical diseases and have a significant global health impact. Accurate identification and quantitation of STH infection is a cornerstone of effective control. Direct observation and counting of eggs in faeces is the current gold-standard method for diagnosis of infection. This approach is time consuming and has poor sensitivity. As ongoing oral benzimidazole therapy across many endemic regions leads to a reduction in STH prevalence and intensity, these sensitivity limitations become an increasingly relevant issue, particularly with respect to monitoring treatment efficacy, identifying reductions in parasite transmission, and accurately quantifying infection burden in discrete populations in middle-income countries. PCR-based detection has long been proposed as an alternative approach to STH diagnosis and many protocols, including quantitative PCR-based methods, have been developed. However, these methods are largely bespoke and use non-standardized reagents that can greatly impact on the transferability and relative consistency of their performance. In the current study, we evaluate an automated, commercially-produced molecular diagnostic tool for validation of the major soil-transmitted helminths, including *Ascaris lumbricoides*, *Trichuris trichiura*, *Necator americanus*, *Ancylostoma duodenale* and *Ancylostoma ceylanicum*, and evaluate its performance in comparison to an established multiplexed qPCR using faecal samples from endemic settings.

## Introduction

Soil-transmitted helminths (STH), including roundworms (*Ascaris lumbricoides*), whipworms (*Trichuris trichiura*) and hookworms (*Necator americanus*, *Ancylostoma duodenale*, *Ancylostoma ceylanicum*) represent a major cause of morbidity in tropical to sub-tropical developing and low-income countries [1]. In 2016, the Global Burden of Disease Study estimated that as many as 3.4 million disability adjusted life years (DALYs) are lost globally due to STH infections each year, of which 1.1, 0.5 and 1.8 million DALYs accounted for roundworm, whipworm and hookworm infections within all age groups in 2015 [2]. Total global infection prevalence is estimated to lie slightly above 1.9 billion infections depending on the diagnostic tool used, and as many as 5.3 billion people are at risk of infection worldwide [3, 4]. Infection risk directly correlates with inadequate sanitation, impoverished conditions, limited access to public health systems and population overcrowding [5]. Symptomology and severity of infection correlates with species of STH and intestinal burden, as well as host age, nutritional and health status [1]. Acute clinical symptoms are less prevalent, but include, for hookworm (*Necator americanus* and *Ancylostoma* spp.) and whipworm (*Trichuris trichiura*), anaemia and diarrhoea, and for roundworm (*Ascaris lumbricoides*), intestinal blockage and/or rupture, leading to ~135,000 deaths per year [6, 7]. Ultimately, the burden of disease caused by morbidity is far more significant than the impact caused by mortality, with long-term sequelae including malnutrition, stunting, wasting and decreased cognitive development [8].

Control of STH infection is dependent on oral anthelmintic therapy using benzimidazoles (BZ) [9]. Currently, the World Health Organization (WHO) recommends, regional mass drug administration (MDA) programs in endemic populations to deliver 400 mg single dose albendazole or 500 mg single dose mebendazole annually or biannually to reduce both infection prevalence and intensity [9]. These MDA programs follow the London Declaration on Neglected Tropical Disease (NTD) 2012 endorsement of the WHO goal to scale up global deworming in order to treat 75% of pre- and school-aged children at least once a year until 2020 in order to decrease STH burden in endemic areas [1]. Quantifying STH burden and the efficacy of BZ MDA programs is dependent on accurate and sensitive diagnosis and quantification of infection, particularly in regions where infection intensity is low but prevalence remains relatively high. Methods used include direct microscopy, formalin-ether concentration method, the McMaster egg counting technique, simple sodium nitrate flotation (SNF) including FLOTAC, the Kato-Katz thick smear (KKTS) and, more recently, PCR-based approaches [10]. The observation and enumeration of STH eggs in faecal samples is the current gold standard for diagnosis of STH infection, with the WHO-recommended, Kato-Katz thick smear the most widely used approach [11]. Advantages of the Kato-Katz method are its cost-effectiveness and its application in remote settings [11]. However, there are several drawbacks of this method, such as the requirement for immediate microscopic examination of multiple freshly collected faecal samples and technicians exhibiting parasitological expertise, a lack of standard protocols (e.g., for the amount of faecal matter examined, fixation method, calculation of eggs per gram faeces) and hence reproducibility, time consumption, labour intensity, the need for rapid assessment of faecal samples to avoid hookworm egg clearance and most importantly the underestimation of infection prevalence due to limited sensitivity [12]. The technique also requires large teams of technicians and equipment to be dispatched to remote communities with an additional requirement for electricity and running water, which is logistically challenging in remote areas. More recently, Inpankaew and colleagues (2014) have established the sodium nitrate flotation method, a direct faecal microscopy-based method used widely in veterinary settings in the past, of which a single application has a 6% higher sensitivity than KKTS (performed in quadruplicate over a two day period to reduce sensitivity limitations) for the detection of hookworm eggs in human stool at any given time point [13].

Copro-microscopic diagnostic methods in combination with regularly administered drug treatment are ideal in highly endemic regions; however, they are not representative in areas containing sub-populations with low prevalence and intensity infections [14]. Further, STH infections are overdispersed, with a majority of the infected harbouring moderate to light intensity infections (roundworm 1–49,999 epg, whipworm 1–9,999 epg, hookworm 1–3,999 epg) and only a minority suffering from high intensity infections (roundworm > 50,000 epg, whipworm > 10,000 epg, hookworm > 4,000 epg) [1, 15]. Consequently, even within highly endemic regions worldwide, the majority of infections may not be readily detectable by microscopy [16, 17]. Although the disease burden in regions of the world has decreased significantly over years of MDA control and increased socioeconomic development, STHs remain a major global human health issue [18]. It is assumed that with successful implementation of MDA treatment a larger decrease in infection intensity than infection prevalence can be observed [19], making the sensitivity limitations of the diagnostic tool a more significant issue than the cost associated with molecular diagnostic approaches [20], as a greater proportion of the population harbours low intensity infections that can potentially be missed by copro-microscopic diagnostic approaches [21]. While these low-grade STH infections may be of a lesser consequence in terms of disease burden, they are highly relevant in terms of any effort to interrupt infection transmission [22], seeing that adults in endemic countries represent infection reservoirs that inhibit the chance of an interruption of transmission cycle by school-based MDA [23]. One major limitation of MDA treatment is the inability to prevent re-infection once treatment has ceased, resulting in rebounding infection levels among targeted communities [24, 25]. Transmission of infection, with a particular focus on populations in low infection intensity settings, needs to be interrupted to stabilize control of STH infections [26], but is influenced by infection prevalence, infection intensity, human migration, regional demography, diagnostic tool application and drug efficacy [27]. Diagnostic tools contribute to reducing transmission by providing a knowledge basis for the specific, targeted and sustainable treatment of sub-populations at risk which serve as a transmission reservoir [28]. Notably, MDA treatment should be administered in combination with other intervention methods to reach satisfactory health outcomes [29]. There is a global need for an appropriate, rapid, cost-effective and sensitive tool for detection of STH infections in order to decrease burden of disease by identifying low-intensity infections and a subsequent targeted sustainable reduction in worm burden [30].

Real-time qPCR methods have recently been tested for STH diagnosis, targeting species-specific gene markers such as ITS-1 or ITS-2 [31–33]. However, these methods currently have limitations that make them not applicable in field settings such as the need for trained scientific personal [31]. Particularly challenging is the transfer of customized qPCR methods among laboratories with a requirement for optimization via significant molecular biological expertise. A reliable, automated diagnostic tool could have the potential to overcome issues related to reproducibility and create a standardized method of STH infection detection.

In the current study, we evaluate a multiplexed-tandem PCR (MT-PCR) based assay to differentiate, identify and quantify each major STH species in genomic DNA isolated directly from stool samples. The method is user-friendly, has high sensitivity and specificity and is produced as a standardized kit that is commercially available and readily transferrable to other laboratories. The method is semi-automated and requires little *a priori* expertise in molecular diagnostics or parasitology. Although unlikely to be cost-effective for routine diagnostics at the present time, the method provides a useful research tool for epidemiological studies of STHs in endemic regions, particularly in populations where prevalence and intensity of infection is highly variable and the limitations of direct egg counting by microscopic examination is impractical or insufficient.

## Methods

### Ethics statement

Written informed consent for this prospective study was received from all study participants, or from parents or guardians for participants under the age of 18 years in Timor-Leste and Cambodia respectively. Ethics approval for the Cambodian based part of this study was provided by the National Ethics Committee for Health Research of the Ministry of Health in Cambodia (269NECHR, 27^th^ of June 2016) as well as by the Human Research Ethics Committee of the University of Melbourne (1647208). Ethic approval for the Timor-Leste based part of this study has been received from the Human Research Ethics Committees at the Australian National University (2015/111) and the Timor-Leste Ministry of Health (2015/196).

### Study areas, faecal sample collection, DNA isolation and multiplexed qPCR validation

#### Timor-Leste

Field sampling structure, sample processing and multiplexed quantitative PCR (qPCR) validation for Timor-Leste samples have been described elsewhere [34, 35]. Briefly, stool samples were obtained from 462 school children attending six primary school in Aileu and Manufahi municipalities, Timor-Leste, at the baseline of the (S)WASH-D for Worms pilot study. A 2–3 g aliquot of each sample was preserved in 5 mL of 5% potassium-dichromate (weight/volume) and transported at room temperature to QIMR Berghofer Medical Research Institute (Brisbane, Australia), where DNA extraction was performed using the PowerSoil DNA isolation kit after the manufacturer’s instructions (Qiagen, Germany). All samples were stored at -20°C following DNA extraction. Initial STH multiplex qPCR targeted *Ascaris lumbricoides*, *Trichuris trichiura*, *Necator americanus* and *Ancylostoma* spp. A total of 462 baseline samples of isolated genomic DNA were used for validation of STH infection prevalence and intensity using the MT-PCR method.

#### Cambodia

Field sampling structure and sample processing of Cambodian samples are as follows. A total of 166 faecal samples from participants originating from ten remote villages in Preah Vihear province, Cambodia were obtained from collaborators in 2016 for testing the MT-PCR method at baseline and 3-month follow-up (n = 332) and had been previously tested by multiplex qPCR targeting hookworm species only using a published protocol (quantification and identification of *Necator americanus*, *Ancylostoma ceylanicum* and *Ancylostoma duodenale*) [36]. Faecal samples were collected in the morning by each participant and transported to the laboratory at room temperature within 60 minutes of sample collection. As much as 3 mL faeces was preserved in 6 mL 5% potassium dichromate for subsequent shipment to the University of Melbourne, Australia. In preparation for DNA isolation, preserved faeces were centrifuged at 2,000 g for 3 minutes, supernatant decanted, and the faecal pellet washed in 15 mL sterile H_2_O (twice) in order to eliminate preservative and avoid interference with downstream molecular assays. 250 mg of the washed faecal pellet was used for DNA isolation using the ISOLATE Faecal DNA Kit (Bioline, UK) according to the manufacturer’s instructions. All samples were stored at -20°C before validation using an established multiplexed qPCR protocol [36]. A total of 302 samples of the cohort described above has been used to validate the developed MT-PCR assay.

### Molecular multiplexed-tandem PCR assay assessment

In collaboration with an industry partner (AusDiagnostics Ptd. Ltd., Australia) we have used an established commercially available multiplexed molecular diagnostic platform, the Easy-Plex system, to develop an assay targeting human STH infections in faecal DNA samples [37]. Multiplexed-tandem polymerase chain reaction (MT-PCR) tests were developed targeting the β-tubulin 1 locus of *Ascaris lumbricoides* (Genbank accession number FJ501301.1), *Trichuris trichiura* (Genbank accession number AF034219.1), *Necator americanus* (Genbank accession number EF392851.1), *Ancylostoma duodenale* (Genbank accession number EF392850.1), and targeting the second internal transcribed spacer region of the nuclear ribosomal RNA gene (ITS-2) for *Ancylostoma ceylanicum* (Genbank accession number JN164660.1). The PCR primers for these assays are held in commercial confidence by AusDiagnostics Pty. Ltd.

The MT-PCR is a nested PCR method consisting of a primary PCR performed in multiplex on a Gene-Plex CAS1212 liquid handling robot (AusDiagnostics, Pty Ltd., Australia), followed by a secondary, tandem real-time qPCR (in which testing is conducted in single-plex in a tandem battery of reactions) run on a LightCycler 480 system (Roche, Switzerland). At the initiation of testing, DNA samples are placed on the Gene-Plex robot deck in a designated loading area and provided a unique sample ID (entered manually into a computer interphase linked to the Gene-Plex robot) which is carried over throughout the MT-PCR testing. Upon initiation of the amplification protocol, the robot distributes 5 μl of each sample DNA to a separate 20 μl reaction well in the “multiplex reaction strip tubes” provided by the STH MT-PCR kit manufacturer. No template controls (dH_2_O) are included with each MT-PCR run and carried over through both amplification phases. Each well of this strip contains lyophilized standardized quantities of each external primer pair for each STH species and an internal spike (consisting of 10,000 copies of a 120 bp, heterologous, synthetic oligonucleotide) to control for PCR inhibition and provide a quantification standard for each sample. The initial multiplex step is performed in a conventional thermocycler unit installed on the Gene-Plex robot deck. This amplification phase consists of 15 cycles with the following parameters: denaturation at 95°C for 10 seconds, annealing at 60°C for 30 seconds and extension at 72°C for 20 seconds with no initial denaturation or final extension phase.

Following the initial multiplex PCR, all first round product amplicons are diluted by the Gene-Plex robot (this is done to eliminate primary carry-over and PCR inhibition) and used as a template for the secondary tandem real-time PCR step. Tandem PCR is conducted on a 384-well plate in 20 μl reactions in a reaction master mix containing SYBR Green I/HRM dye. Each well of the 384-well plate contains lyophilized internal PCR primers for one target species or the internal spike control per reaction arranged in tandem array (i.e., multiplex amplicon from one sample is loaded onto a tandem array of six consecutive reaction wells, with each well containing one amplicon specific primer pair). Following loading of the tandem PCR plate, the plate is sealed using a self-adhesive heat-adherent film (MSB1001, BioRad, USA) and then loaded onto a LightCycler 480 system (Roche, Switzerland). The secondary amplification consisted of 30 cycles with the following profile: denaturation at 95°C for 10 seconds, annealing at 60°C for 15 seconds and extension at 72°C for 15 seconds with initial denaturation at 95°C for 10 minutes and no final extension phase.

Following the tandem real-time PCR phase, each amplicon is subjected to high-resolution melt-curve (HRM) analysis (from 72°C to 95°C). Following HRM, the melt profile for each amplicon for each sample is assessed for quality, purity (based on melt peak number, height and width) and specific identity (based on estimated melting temperature relative to control parameters determined using purified positive control DNAs for each targeted species during initial assay development by AusDiagnostics Pty. Ltd.). A positive or negative test call was determined based on these HRM results. Quantity of each test-positive amplicon was then calculated based on its amplification cycle threshold (Ct-value) relative to the cycle threshold of the sample-specific internal spike control. From the initial loading of the sample DNAs onto the Gene-Plex robot, all steps of the MT-PCR method are automated, including the determination of positive test results and their quantitation, which is provided at the end of the reaction in a tabular and graphical format by the software (MT Analysis Software, AusDiagnostics Pty. Ltd., Sydney) used to run the reaction protocol (MT Assay Setup Software for the multiplex-PCR phase, AusDiagnostics Pty. Ltd., Sydney; and LightCycler 480 Software Version 1.5 for the tandem-PCR phase, Roche, Switzerland). Upon initiation of the initial multiplex reaction protocol, the only additional operator input required prior to final test results is the application of a heat-sealing film between the multiplex and tandem-PCR phases and the transfer of the sealed tandem-PCR reaction plate from the Gene-Plex robot to the Lightcycler.

### Evaluation of MT-PCR diagnostic performance

All samples tested in the current study were assessed previously for STH infections by multiplex qPCR [34, 35]. We evaluated both the quantitative and qualitative diagnostic performance of the MT-PCR method against the previous molecular test results. Run-to-run variation was assessed for the assay by testing all 462 (5 μl) faecal DNA samples from Timor-Leste in duplicate, with disagreements (n = 31; 6.7%) tested in triplicate. As this testing showed high run-to-run consistency between replicates, subsequent testing of samples from Cambodia (n = 302) has been performed in single replicate (5 μl). Diagnostic sensitivity of the MT-PCR was assessed using the previously published multiplex qPCR as the diagnostic gold standard; i.e., we defined a “true positive” as samples that are positive by both molecular diagnostic methods (multiplex qPCR and MT-PCR) and a “true negative” as samples that are negative by both methods. MT-PCRs that disagreed with the multiplex qPCR were defined as “false positive” or “false negative” respectively. These potentially “false positive” or “false negative” samples were restested via the previously published conventional qPCR with slight modifications. Briefly, we tested all disagreements using a single-plex qPCR approach for the respective gene targets (*Ascaris lumbricoides* n = 31, *Ancylostoma ceylanicum* n = 1, *Trichuris trichiura* n = 1 and *Necator americanus* n = 25). Tests were evaluated using 1x SensiFAST SYBR No-ROX mastermix (Bioline, UK), 2 μl template DNA and optimized primers as described elsewhere in a total reaction volume of 20 μl [32, 35]. The DNA quantification and melt-curve analysis for all tests was performed on the LightCycler 480 instrument (Roche, Switzerland) using the following conditions: 3 minutes at 95°C followed by 40 cycles of 9 seconds at 95°C and 30 seconds at 60°C with a final step-wise denaturation from 60°C to 97°C in 1.1°C/second increments. On retesting, we consistently found primer dimer formation at Ct values equal to 35 (n = 18) within the negative control of the *N*. *americanus* qPCR test after 35 cycles. For the purpose of verifying that sample degradation had not influenced MT-PCR testing, we considered any single-plex qPCR retests as negative if amplification was detected above Ct = 35.

### Data analysis

Data and statistical analysis was performed using the Stata 12.1 (StataCorp LP, USA), Prism 7.0b (Graphpad Software Inc., USA), RStudio 1.1.463 (RStudio, Inc., USA) and Excel 15.4 software (Microsoft, USA). Power calculations for chi-squared tests were performed in RStudio 1.1.463 (RStudio, Inc., USA) using the *pwr* package 1.2–2 [38]. All tests used a significance level of p = 0.05 with a degree of freedom (df) of 2–1 (i.e., 1) (S3 Table). STH infection prevalence and intensity data were analysed in Excel 15.4 with visualization in Prism 7.0b. Interrater agreement values—described as Cohen’s κ—between both molecular diagnostic tests, 95% confidence intervals, significance of κ (using a p-value threshold for statistical significance of 0.05), sensitivity and specificity of MT-PCR have been determined using the Stata 12.1 software package. Cycle threshold values of MT-PCR vs qPCR were plotted against each other in a scatterplot and a simple linear regression analysis was performed using Prism 7.0b with R^2^ values confirmed through regression analysis in Stata 12.1.

## Results

**Fig 1:**
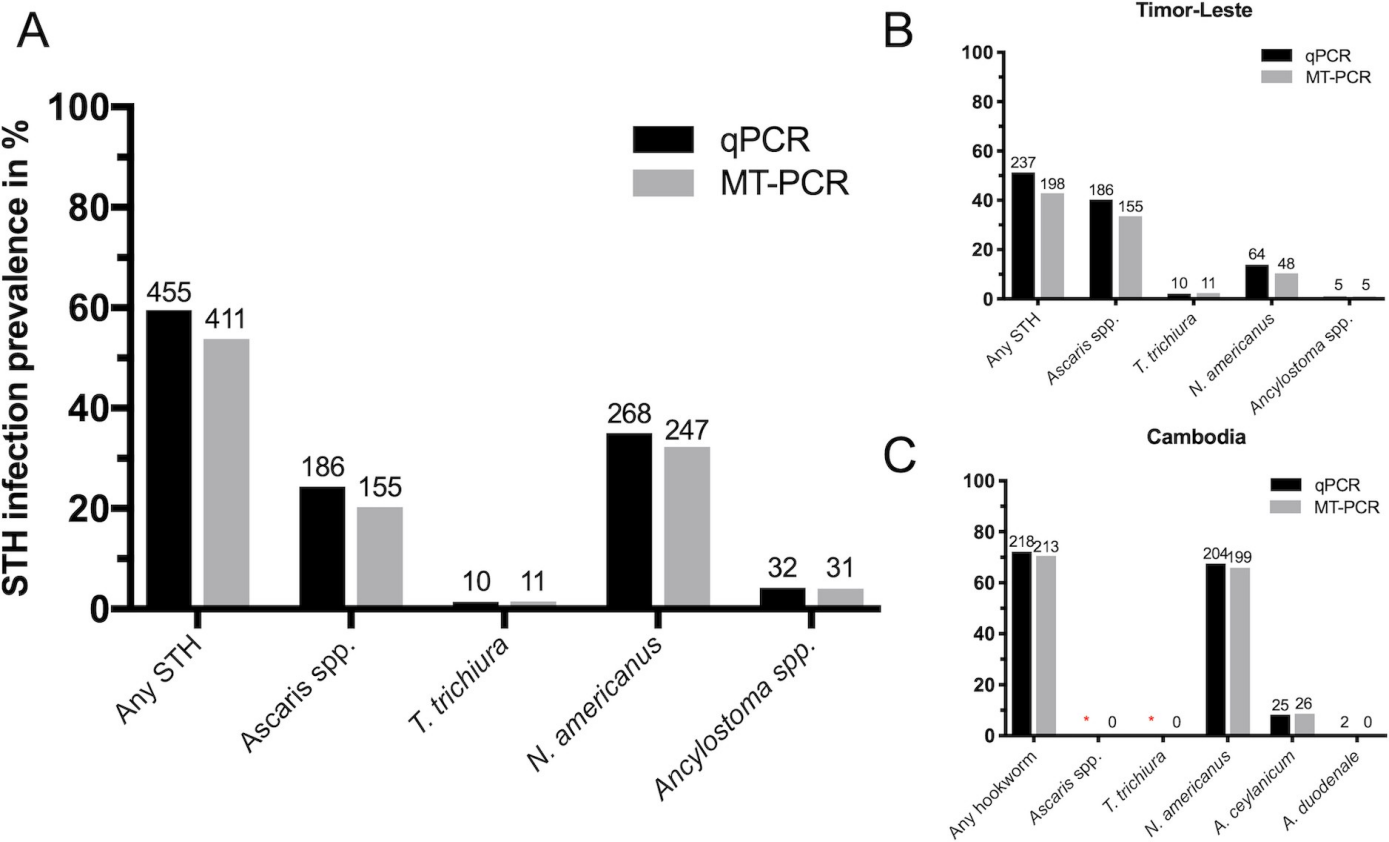
**Overall infection prevalence of the major STH species comparing multiplexed qPCR and MT-PCR diagnostic approaches in cohorts from Timor-Leste (B) Cambodia (C) and combined (A).** (A) Representation of combined infection prevalence for Timor-Leste and Cambodian cohort in percent with total numbers of positive infections as seen on individual bars. (B) Estimation of infection prevalence in Timor-Leste cohort of 462 stool samples with percentage infection prevalence on y-axis and STH species by diagnostic method on x-axis. (C) Estimation of infection prevalence in Cambodian cohort of 302 stool samples with percentage infection prevalence on y-axis and STH species by diagnostic method on x-axis. Estimation of infection by qPCR has been conducted for hookworm infections only. Field study setting is known to show very little to no *A*. *lumbricoides* or *T*. *trichuris* infections as to why these infections have not been included in estimation of infection prevalence by qPCR with confirmation of zero positive infections by MT-PCR within the scope of this study (*).

This study evaluated a total of 764 faecal DNA samples (462 Timor-Leste and 302 Cambodia) that had been previously tested by qPCR examination [34, 35]. Each sample was tested by MT-PCR as described above, of which 20.3% were positive for *Ascaris lumbricoides*, 36.4% were positive for hookworm (32.3% were positive for *Necator americanus*, 4.1% *Ancylostoma ceylanicum*) and 1.4% were positive for *Trichuris trichiura*. No infections were detected for *Ancylostoma duodenale*. Infection prevalence in Timor-Leste with any soil-transmitted helminth was 42.9% with species-specifc infections of 33.5% for *A*. *lumbricoides*, 2.4% for *T*. *trichiura*, 10.4% *N*. *americanus* and 1.1% *Ancylostoma ceylanicum*. Hookworm infections dominated in the Cambodian cohort with prevalence of 65.9% for *N*. *americanus* and 8.6% for *Ancylostoma ceylanicum* infections. Overall infection prevalence with any STH species was generally higher in Cambodia (70.5%) than Timor-Leste (42.9%) (Fig 1).

Chi-square table values for all investigated species are as follows: *A*. *lumbridcoides* 276 true negative, 31 false negative, 0 false positive, 155 true positive (n = 462); *N*. *americanus* 494 true negative, 23 false negative, 2 false positive, 245 true positive (n = 764); *T*. *trichuris* 451 true negative, 0 false negative, 1 false positive, 10 true positive (n = 462); *Ancylostoma* spp. 732 true negative, 1 false negative, 0 false positive and 31 true positive (n = 764). Total diagnostic infection values for any STH species are 307 true negative, 46 false negative, 2 false positive and 409 true positive (n = 764) as shown in Table 1.

**Table 1:** 2x2 contingency table for any STH infections. Any STH infections is defined as a positive infection by molecular diagnostic analysis with one or more STH species.

	MT-PCR positive	MT-PCR negative	Total
**qPCR positive**	409	46	455 (59.55%)
**qPCR negative**	2	307	309 (40.45%)
**Total**	411 (53.80%)	353 (46.20%)	764

**Fig 2:**
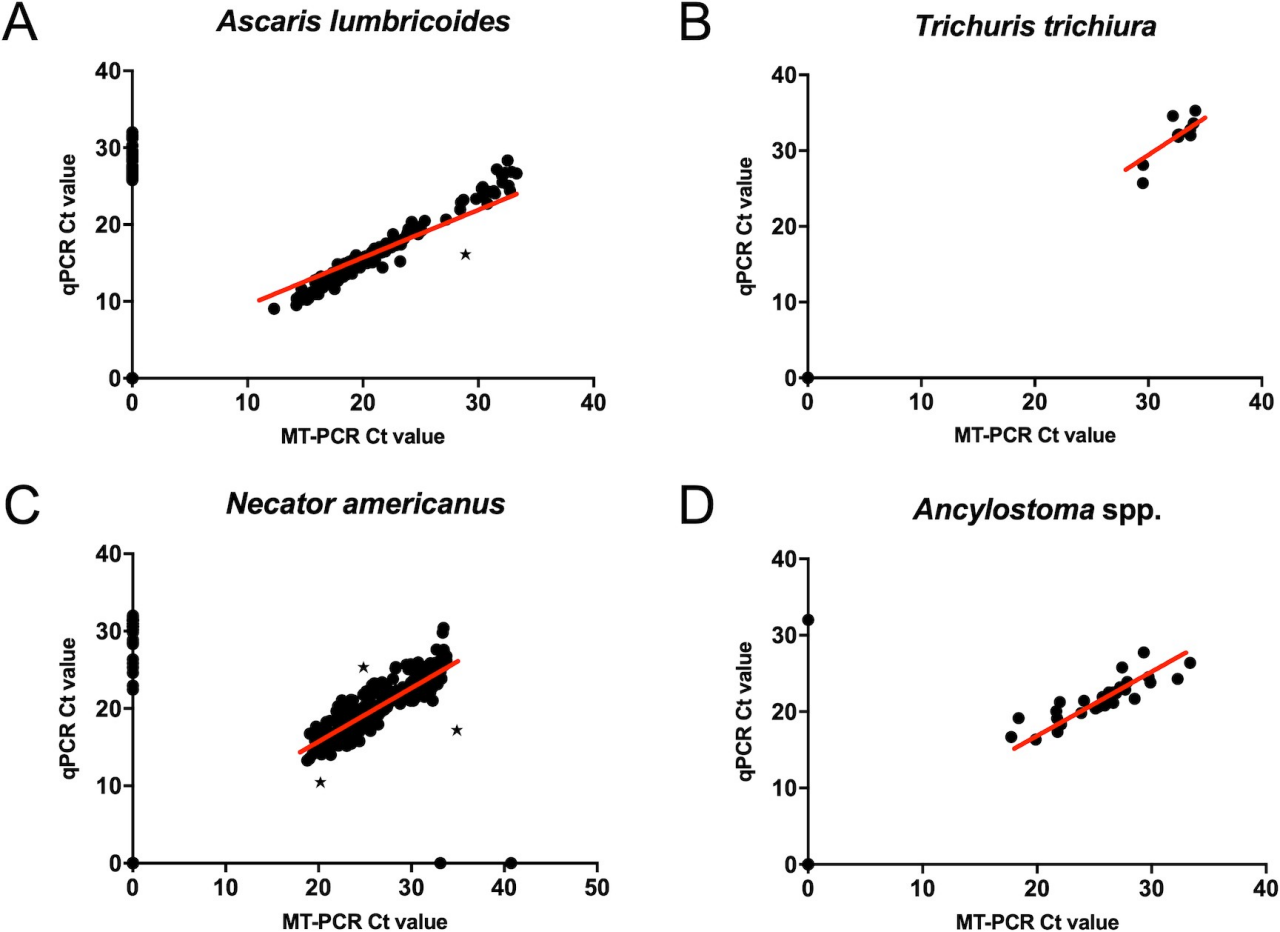
Cycle threshold (Ct) value scatterplot for all investigated STH species showing agreement of multiplexed qPCR and MT-PCR for all samples tested infection positive by either none, one or both molecular diagnostic methods. *Ascaris lumbricoides* (A) *Trichuris trichiura* (B) *Necator americanus* (C) *Ancylostoma* spp. combined values for *A*. *duodenale* and *A*. *ceylanicum* (D). Highlighted samples were removed for analysis of coefficient of determination which determines the closeness of data to a fitted linear regression line using only samples deemed infection positive by both molecular diagnostic methods (*).

Interrater reliability values, kappa (κ), showed a very good agreement between MT-PCR and conventional qPCR (Table 2), as defined by Cohen [39], as follows: <0.2 poor agreement, 0.20–0.40 fair agreement, 0.40–0.60 moderate agreement, 0.60–0.80 good agreement and 0.80–1.00 very good agreement. Total amount of agreement between both molecular diagnostic methods for true positive and true negative validation shows a percentage of agreement for all tests > 93.29%. Diagnostic specificity (true negative rate) of the MT-PCR method relative to multiplex qPCR was above 99.60% for all tests (Table 2). Diagnostic sensitivity (true positive rate) relative to multiplex qPCR ranged from 83.33% (*Ascaris*) to 100.00% (*Trichuris*) with three tests above 91.42%. The quantitative capacity of the MT-PCR method was compared by linear correlation of MT-PCR vs qPCR considering samples that were infection positive by both diagnostic methods (Table 2, Fig 2). Quantitative capacity was visualized considering all samples independent of infection prevalence by the various diagnostic methods (Fig 2). MT-PCR gene copy number estimates ranged from 28–70,059,042 with a median of 590,956.3 for *A*. *lumbricoides*, 41–1145.5 with a median of 84.5 for *T*. *trichura* and 15–781,465 for hookworms, with species-specific medians of 4228 for *N*. *americanus* and 4940 for *A*. *ceylanicum*, providing a possible initial starting point of inferring infection intensity measure/intestinal burden from gene copy number counts.

**Table 2:** Interrater reliability as defined by kappa agreement values (Cohen’s kappa, κ) including standard error of κ, 95% confidence interval (CI) of κ and significance of κ; and coefficient of determination (R^2^) for qPCR vs MT-PCR to determine closeness of data to fitted linear regression line. Standard errors (SE) range from 0.0361 to 0.0465 with a 95% confidence interval ranging from 0.808 to 1.00 among all tests. All p-values for κ are <0.00001 assuming interrater reliability measurements to be significant. R^2^ values are given in percentages of the total number of samples validated for each STH species (N). Only samples deemed infection positive by both methods were included in this analysis (1 *Ascaris lumbricoides* and 3 *Necator americanus* outliers were removed (highlighted in Fig 2)). All *Ancylostoma ceylanicum* positive infections represented co-infections with *Necator americanus* and were subsequently discarded from this analysis.

	Total Agreement (% Agreement)	Sensitivity (%)	Specificity (%)	κ	SE of κ	95% CI of κ	p-value of κ	N	qPCR vs MT-PCR R^2^ (%)
***A*. *lumbricoides***	431 (93.29)	83.33	100.00	0.8566	0.0460	0.808–0.905	p<0.00001	155	97.36
***T*. *trichiura***	461 (99.78)	100.00	99.78	0.9513	0.0465	0.856–1.000	p<0.00001	10	75.48
***N*. *americanus***	739 (96.73)	91.42	99.60	0.9268	0.0361	0.899–0.955	p<0.00001	245	80.63
***Ancylostoma* spp.**	763 (99.87)	96.88	100.00	0.9834	0.0362	0.951–1.000	p<0.00001	-	-

## Discussion

We have evaluated the multiplex-tandem PCR as a semi-automated diagnostic tool suitable for human STHs, comparing its performance to a multiplexed qPCR assay. Although copro-microscopic analysis is overwhelmingly the predominant method for diagnosis of STH infections, there is no clear gold standard for determining “true positives” in STH diagnostics [22, 40]. Based on this, we adopted the approach of considering a “false positive” or “false negative” by MT-PCR based on previous multiplex qPCR results for this dataset [34, 35], followed by re-testing of the disagreeing samples using a single-plex qPCR with an established ITS-1 or ITS-2 gene marker (Genbank accession numbers AB571301.1, FM991956.1, AJ001599.1, EU344797.1) [35]. Using this approach, two “false positive” tests (n = 2) in the MT-PCR validation (n = 764) determined relative to qPCR were confirmed as true positives, yielding a total diagnostic specificity of above 99.78% for each species assay.

We note that all faecal samples tested in the current study are samples collected from field sites in Timor-Leste and Cambodia in 2016 and have been stored at -20°C since their original testing by qPCR. After several freeze-thaw cycles, it is possible that the samples were influenced by DNA degradation, which may influence their subsequent detection. To test this, we retested all samples that were “false negative” by MT-PCR (n = 55), using the conventional qPCR protocol they originally tested positive by. Per the above, 38 of these samples retested as positive with an average Ct value of 30.80 (S1 Table). The remaining 17 samples yielded faint positives with the majority of Ct values between 30 to 35 (S1 Table). This indicates that *Ascaris lumbricoides* MT-PCR has a slightly lower sensitivity than the conventional qPCR, but that sample degradation may have impacted on some of our test results, and requires further evaluation with fresh field samples. Using this approach, total diagnostic sensitivity of the MT-PCR ranged from 90.64 (*Ascaris*) to 100.00% (*Trichuris*).

Overall, our study supports recent efforts to develop qPCR as a diagnostic alternative to or complement of faecal microscopy [41]. Each MT-PCR assay had very good agreement (kappa > 0.85) and strong quantitative correlation (R^2^ > 0.7548) to a recently published multiplexed qPCR for these species [35, 36]. Limitations of the quantitative correlation include limited numbers of *T*. *trichuris* positive samples (n = 10) as well as no *Ancylostoma* spp. single infection positive samples, which are required to present a complete assay evaluation (Table 2). Both standard qPCR and the MT-PCR methods have clear advantages over faecal microscopy in terms of sensitivity. The limited sensitivity of faecal microscopy for STH detection, particularly hookworm, is well documented in the literature [42, 43]. This has increasingly been noted as a challenge to sustainable STH control in regions where prolonged oral MDA programs have resulted in a substantial reduction in worm burden such that the majority of infections now fall below the WHO definition for “light intensity” infections [1]. This reduction will clearly impact on the global STH disease burden and with the increased economic development in many STH endemic countries as well as the potential for the emergence of drug resistant helminths, the focus needs to begin to shift toward sustainable control and STH transmission interruption [44]. Programs to monitor transmission and to allow efficient control of STHs in populations where regional MDA programs are no longer sensible will require more sensitive diagnostic approaches than faecal microscopy can provide [21]. As shown in previous studies of parasitic nematode infections, nested qPCR diagnostics show a higher sensitivity measure compared to that of a standard qPCR while including a quantitative estimation missing in traditional PCR approaches [45].

The primary limitations associated with a shift to a molecular or PCR-based diagnostic for STH infections include (i) the complexity of PCR application in endemic settings, (ii) a limitation in the transferability or reproducibility of bespoke PCR techniques and a need for standardization for clinical applications, (iii) the lack of clear relatability (conversion of molecular diagnostic Ct values to epg counts) of PCR-based test results to WHO treatment/burden guidelines, which is based on faecal egg densities and found to be on average 4x lower than egg intensity counts by molecular diagnostic for *Necator americanus* [36], and (iv) the relative cost differences between microscopic and PCR-based detection. The MT-PCR method developed here is built around a user-friendly, largely automated robotic platform that requires minimal molecular biological expertise. All kit reagents are produced and standardized by a commercial entity accredited for clinical diagnostic assay production, supporting rapid and reliable transfer among laboratories. The MT-PCR method is quantitative and further evaluation of the correlation between copy numbers and eggs per gram will provide meaningful information on helminth intensity as well as assist in its translation to assess intensity-related morbidity. However, as found in other studies investigating the use of multiplexed qPCR for STH diagnosis [40], correlations between qPCR results and egg intensities are moderate. These results can be inferred via logistic regression, but lack of knowledge about target gene copy number per genome limits our ability to make conclusions about helminth burden (particularly for loci, such as the nuclear ribosomal RNA gene, that are of an unstable copy number) [46]. Establishing these threshold levels will require additional screening of well characterized control samples of known egg densities as well as further field evaluation including patient specific health records to assess burden.

Finally, regarding cost, recent estimates for multiplex qPCR-based testing of STH infected stools put the cost per sample at US$2.61 [32] as a single-well and qPCR mastermix are used per sample (run in duplicate). The same study estimated true costs per sample for microscopy at US$2.60, indicating that the cost-effectiveness of microscopy is likely over-estimated. At present, the MT-PCR method is not competitive with these costs, with testing costing approximately US$7.31 (AUS$10.17) per sample for the current assay configuration following DNA extraction (this calculation does not include cost of liquid handling robot or qPCR thermocycler; approximate cost of DNA extraction US$ 6.85 (AUSD$ 9.64)). However, our focus here was on assay evaluation not on maximizing the efficiency or economics of the system. Overall, we acknowledge the current limitation of the platform, considering all resources associated, to laboratory-based most likely non-endemic settings. We have addressed the issue of to date limited data availability of PCR based helminth diagnostics approaches which perform with an increased sensitivity compared to currently performed copro-microscopic approaches [22].

In summary, we find the MT-PCR method to be a rapid, semi-automated and user-friendly molecular diagnostic tool for STH infection that provides comparable performance to conventional multiplex qPCR and superior sensitivity to faecal microscopy.

## Supporting information

S1 Table Raw data file of MT-PCR and qPCR diagnostics. (XLSX)

S2 Table STARD checklist. (DOCX)

S3 Table Statistical power analysis. (DOCX)

S1 Fig STARD flow-diagram. (DOCX)
