# Supplementary material for: Development and validation of a multiplexed-tandem qPCR tool for diagnostics of human soil-transmitted helminth infections
Source: PLoS Negl Trop Dis. 2019 Jun 17;13(6):e0007363. doi: 10.1371/journal.pntd.0007363 (PMC6597125; doi:10.1371/journal.pntd.0007363)
Supplement: S3 Table — (DOCX) [file pntd.0007363.s003.docx]

|  | N | Small effect  (w = 0.1) | Medium effect  (w = 0.3) | Large effect  (w = 0.5) |
| --- | --- | --- | --- | --- |
| *A. lumbricoides* | 764 | 0.789 | 1 | 1 |
| *T. trichiura* | 764 | 0.789 | 1 | 1 |
| *N. americanus* | 462 | 0.575 | 0.999 | 1 |
| *Ancylostoma* spp. | 462 | 0.575 | 0.999 | 1 |
