# Supplementary material for: Development and validation of a multiplexed-tandem qPCR tool for diagnostics of human soil-transmitted helminth infections
Source: PLoS Negl Trop Dis. 2019 Jun 17;13(6):e0007363. doi: 10.1371/journal.pntd.0007363 (PMC6597125; doi:10.1371/journal.pntd.0007363)
Supplement: S1 Fig — (DOCX) [file pntd.0007363.s004.docx]

Enrolled study participants

n= 1492 *CA*

No stool submitted

n= 257  *CA*

Eligible participants for STH screening

n= 1235 *CA*

Non-eligible STH negative study participants

n= 1069 *CA*

Potentially eligible STH positive study participants

n= 166 (+ 166 follow-up) *CA*

No stool submitted (follow-up)

n= 27  *CA*

Eligible STH positive study participants

n= 166 (+ 159 follow-up)  *CA*

Potentially eligible participants

n= 522 *TL*

No stool submitted

n= 60 *TL*

Multiplex qPCR validation

n= 787  *TL+CA*

No sample material left after microscopy and multiplex qPCR

n= 23 *TL+CA*

MT-PCR validation

n= 764 *TL+CA*

**Final diagnosis - infection negative**

*Ascaris lumbricoides* (n= 307)

*Trichuris trichiura* (n= 451)

*Necator americanus* (n= 517)

*Ancylostoma duodenale* (n= 764)

*Ancylostoma ceylanicum* (n= 733)

Inconclusive (n= 0) *TL+CA*

**Final diagnosis - infection positive**

*Ascaris lumbricoides* (n= 155)

*Trichuris trichiura* (n= 11)

*Necator americanus* (n= 247)

*Ancylostoma duodenale* (n= 0)

*Ancylostoma ceylanicum* (n= 31)

Inconclusive (n= 0) *TL+CA*

No MT-PCR validation

n= 0 *TL+CA*

multiplex qPCR standard

n= 411 *TL+CA*

multiplex qPCR standard

n= 353 *TL+CA*

No qPCR standard

n= 0 *TL+CA*

MT-PCR positive

n= 411 *TL+CA*

MT-PCR negative

n= 353 *TL+CA*
